# Genomic Validation in the UK Biobank Cohort Suggests a Role of C8B and MFG-E8 in the Pathogenesis of Trigeminal Neuralgia

**DOI:** 10.1007/s12031-024-02263-x

**Published:** 2024-10-03

**Authors:** Muataz S. Lafta, Gull Rukh, Sami Abu Hamdeh, Yasmina Molero, Aleksandr V. Sokolov, Elham Rostami, Helgi B. Schiöth

**Affiliations:** 1 Department of Surgical Sciences, Functional Pharmacology and Neuroscience, Uppsala University, Uppsala, Sweden; 2 Department of Medical Sciences, Neurosurgery, Uppsala University, Uppsala, Sweden; 3 Department of Neuroscience, Karolinska Institutet, Stockholm, Sweden

**Keywords:** Trigeminal neuralgia, Proteome, Independent genetic variants, UK Biobank

## Abstract

Trigeminal neuralgia (TN) is a severe facial pain disease of uncertain pathophysiology and unclear genetic background. Although recent research has reported a more important role of genetic factors in TN pathogenesis, few candidate genes have been proposed to date. The present study aimed to identify independent genetic variants in the protein-coding genes associated with TN. We focused on genes previously linked to TN based on the results of four proteomic studies conducted by our research team. The goal was to validate these findings on the genetic level to enhance our understanding of the role of genetics in TN. The study is based on the participants from UK Biobank cohort. Following quality control, 175 independent single nucleotide polymorphisms (SNPs) in 17 genes were selected. The study sample comprised of diagnosed TN cases (N = 555) and randomly matched controls (N = 6245) based on specific criteria. Two SNPs corresponding to *C8B* rs706484 [odds ratio (OR) (95% confidence interval (CI)): 1.357 (1.158–1.590); p: 0.00016] and *MFG-E8* rs2015495 [OR (95% CI): 1.313 (1.134–1.521); p: 0.00028] showed significant positive association with TN, indicating a positive effect of the SNP alleles on gene expression and disease risk. Interestingly, both SNPs are Expression Quantitative Trait Loci (eQTLs), and are associated with changes in the expression activity of their corresponding gene. Our findings suggest novel genetic associations between C8B, a key component of the complement system, and MFG-E8, which plays a role in regulating neuroinflammation, in relation to TN. The identified genetic variations may help explain why some individuals develop TN while others do not, indicating a potential genetic predisposition to the condition.

**Supplementary Information:**

The online version contains supplementary material available at 10.1007/s12031-024-02263-x.

## Introduction

Trigeminal neuralgia (TN) is a chronic neuropathic pain condition originating from the trigeminal nerve, the fifth cranial nerve. Recognized as the most common and severe facial pain syndrome, TN is characterized by recurrent stabbing, paroxysmal, or burning pain in the orofacial region (Bista & Imlach 2019, Jones et al. 2019, Gambeta et al. 2020). In the majority of cases, TN may arise from vascular compression on the trigeminal nerve root, termed classical TN. Alternatively, it may result from lesions affecting the trigeminal nerve, such as tumors, vascular malformations, or multiple sclerosis, termed secondary TN. A third subtype, known as idiopathic TN, lacks an identified cause. While the leading theory for the underlying cause of TN implicates neurovascular compression (NVC), typically by the superior cerebellar artery, causing demyelination and resulting in ectopic firing, the existence of TN in patients without NVC and the presence of NVC in asymptomatic individuals has warranted a search for alternative explanation models for this condition (Montano et al. 2015, Devor et al. 2002, Panchagnula et al. 2019, Leal et al. 2014).

While the majority of TN cases are considered sporadic, familial TN is estimated to make up between 2 and 11% of cases (Fernández Rodríguez et al. 2019, Familial Trigeminal Neuralgia 2020), with the etiology remaining unknown for both forms (Gambeta et al. 2020, Smyth et al. 2003, Denu et al. 2017, Harsha et al. 2012). Despite the unclear role of genetic factors in TN development, recent systematic reviews on TN and genetics have indicated a more significant involvement of genetic factors in TN pathophysiology than previously assumed (Panchagnula et al. 2019, Alsaloum et al. 2020, Smith et al. 2021, Doshi et al. 2020). Studies on familial TN have revealed earlier onset in patients compared to those with sporadic TN (Mannerak et al. 2021, Méreaux et al. 2019). The inheritance patterns are unclear, with some studies suggesting autosomal dominant inheritance and others demonstrating autosomal recessive patterns (Fernández Rodríguez et al. 2019, Cervera-Martinez et al. 2018). Despite this evidence, comprehensive genetic linkage studies, genome-wide association studies (GWAS), or next-generation sequencing studies for TN have not been reported to date (Panchagnula et al. 2019).

Even though there are relatively few human studies on the role of genetic factors in TN, several candidate genes have been proposed. The genetic background of TN appears to be complex, involving multiple genes, specifically those coding for sodium channels (Dong et al. 2020, Tanaka et al. 2016, Stefano et al. 2020), calcium channels (Gambeta et al. 2020, Stefano et al. 2020), and serotonin transporters (Cui et al. 2014). Several genes have been suggested in human studies that may contribute to the development of TN (Mannerak et al. 2021). On the other hand, there is a growing body of studies on experimental trigeminal pain in animals that have identified a number of candidate genes, primarily involved in different ion channels, purinoceptors, and tachykinin proteins (Lulz et al. 2015, Pathophysiological 2024, Benedet et al. 2017, Trigeminal Injury Causes kappa Opioid-Dependent Allodynic 2010, Ma et al. 2012).

Advancements in protein analysis technology provide new opportunities for simultaneous high-throughput investigations of genes. In previous research, we conducted four proteomic studies to investigate the molecular mechanisms underlying the pathogenesis of TN (Ericson et al. 2019, Abu Hamdeh et al. 2020, Svedung Wettervik et al. 2022, Lafta et al. 2023), implicating a total of 17 proteins with TN based on the key findings from each study. The objective of this study was to investigate if there is genetic evidence in the UK Biobank (UKB) supporting the role of these proteins in TN. We collected the SNPs associated with the genes corresponding to the 17 proteins and investigated the association between each SNP and TN using UKB data, aiming to identify independent genetic variants for TN.

## Materials and Methods

### United Kingdom Biobank (UKB)

The current study was based on data acquired from the UKB resource, which is a large population-based cohort with extensive health-related data on more than 500,000 individuals, aged 40 to 69 years, with an almost equal distribution between men and women (Sudlow et al. 2015).

All the participants provided written informed consent for their data to be used in future research with the possibility to withdraw at any time and the UK Biobank project has been approved by the UK Biobank Research Ethics Committee (REC), (REC reference 11/NW/0382). This study was conducted using the UK Biobank Resource under application number 30172 and for our use of UK Biobank data, an approval was obtained by the Regional Ethics Committee of Uppsala, Sweden (2017/198).

### Genotype Data

#### Included Studies for Gene Selection

A summary of the steps to create the genotype data is shown in Flowchart 1. We focused on genes with potential functional association with TN. Based on the results of four previous proteomic studies conducted by our research group (Ericson et al. 2019, Abu Hamdeh et al. 2020, Svedung Wettervik et al. 2022, Lafta et al. 2023), we aimed to validate these findings on the genetic level to enhance our comprehension of the role of genetics in TN. Of these four studies, three utilized Olink Proximity Extension Assay technology, while the fourth employed in-depth mass spectrometry. We compiled the key findings from each study, specifically the proteins and their corresponding genes, resulting in a total of 17 genes.

**Flowchart. 1:**
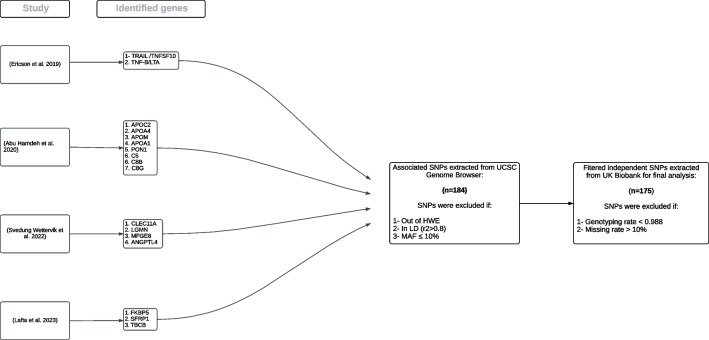
Flowchart showing the steps for the generation of the genotype data. Abbreviations: SNP, single nucleotide polymorphism; LD, linkage disequilibrium; HWE, Hardy–Weinberg Equilibrium; MAF, minor allele frequency

In the first study by Ericson et al. (Ericson et al. 2019), a panel of 92 protein biomarkers related to inflammation in lumbar cerebrospinal fluid (CSF) from patients with TN (n = 27) was compared to individuals without TN. Two proteins, TRAIL and TNF-β, were identified as being of specific interest for TN. In the second study by Abu Hamdeh et al. (Abu Hamdeh et al. 2020), lumbar CSF from TN patients (n = 17) and from controls was analyzed using in-depth mass spectrometry. The study identified apolipoproteins (APOC-2, APOA4, APOM, APOA1, and PON1) and proteins involved in the complement system (C5, C8B, and C8G) as elevated and significantly over-represented. In the third study by Wetterwik et al. (Svedung Wettervik et al. 2022), differences in protein expression of 91 CSF proteins of TN patients (n = 16) in relation to controls were explored. The TN patients exhibited higher concentrations of Clec11a, LGMN, MFG-E8, and ANGPTL-4 in CSF than the controls. And lastly in the fourth study by Lafta et al. (Lafta et al. 2023), protein expression levels in both serum (n = 33) and CSF (n = 27) were explored in TN patients compared to multiple sclerosis (MS) patients and controls. The main findings included three proteins related to neuroinflammation (SFRP1), chronic stress (FKBP5), and neurodegeneration (TBCB) in TN patients.

### Filtering Steps for SNP Selection

The UCSC Genome Browser database was used to identify all the SNPs associated with each of these selected 17 genes. The UCSC Genome Browser, hosted by the University of California, Santa Cruz (UCSC), serves as a comprehensive open-access knowledge platform, accessible both online and for download (Rosenbloom et al. 2012). The associated SNPs were collected from the latest release of dbSNP153 obtained from the UCSC Genome Browser. Gene coordinates were obtained for each gene using the longest corresponding transcript from NCBI RefSeq track. Genome assembly GRCh37/hg19 was used in all steps.

We applied a tool, *bedToBigBed* from UCSC, to extract SNPs based on specified genetic coordinates solely within the complete length of the gene, without any base pairs located outside the gene's boundaries. The obtained list of SNPs was then further filtered to include only SNPs with a minor allele frequency (MAF) of 0.1 or higher in the 1000 Genomes Project. We chose this high allele frequency threshold based on evidence that loci with a low MAF (< 10%) have significantly lower power to detect weak genotypic risk ratios than loci with a high MAF (> 40%) (Ardlie et al. 2002). Furthermore, previous studies have demonstrated that rare genotypes are more likely to result in spurious findings (Lam et al. 2007).

Lastly, to identify independent variants, specifically SNPs that are not in linkage disequilibrium (LD) with each other, we analyzed one gene at a time and examined the LD within each SNP pair using r2 as a measure of LD (SNPs with r2 < 0.8 were selected). LD is the statistical correlation between alleles at different loci, providing valuable information about the evolutionary forces affecting a population as well as the genetic basis of complex traits and diseases (Slatkin 2008). We used an R package, *LdlinkR* (Myers et al. 2020), to prune the list of SNPs by LD on a chromosomal basis with r2 set to 0.8, MAF set to 0.1, and the default CEU population. These filtering steps yielded a total of 184 independent SNPs for our 17 genes. Finally, we extracted the genotype data for each SNP from the UKB, using two different filters to exclude SNPs with genotyping rate < 0.988 and/or missing rate > 10%. This resulted in a final number of 175 SNPs that were used in the analyses.

### Phenotype Data

A summary of the steps to create the phenotype data is shown in Flowchart 2. In the UKB, phenotypic data was available for 502,696 participants. We excluded participants who had withdrawn their consent, were genetically related (UKB FID: 22021), or were non-European (UKB FID: 21000), which yielded a final number of 292,161 participants. The cases for TN were identified using the International Classification of Diseases (ICD-10) code G50.0 for TN. Participants were determined as cases if they either had a primary or secondary diagnosis of TN from linked hospital admission records, which yielded a total of 555 cases.

**Flowchart. 2:**
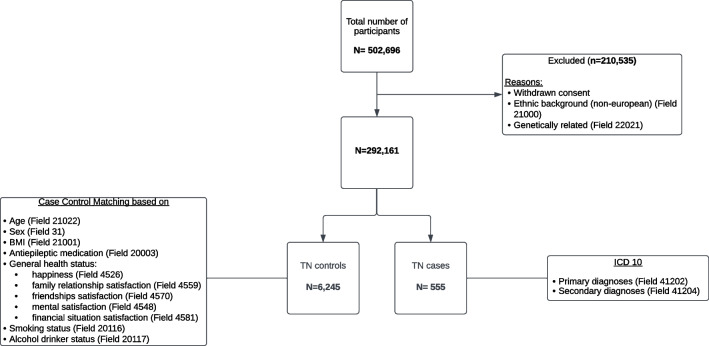
Flowchart showing the selection of study samples for trigeminal neuralgia (TN) cases and controls

The controls were derived from participants who did not meet the above-mentioned criteria for the definition of cases. We utilized a case–control matching procedure in SPSS, randomly pairing cases with controls based on criteria known to affect the incidence and prevalence of TN. This method also facilitated the examination of the effects of SNPs on TN, ensuring that the comparison between groups was robust and accounted for potential confounding factors that could influence our study's findings. We selected controls based on age (UKB field ID (FID): 21022), sex (UKB FID: 31), BMI (UKB FID: 21001), antiepileptic medication (UKB FID: 20003), alcohol drinker status (UKB FID: 20117), smoking status (UKB FID: 20116), and general health status, including happiness (UKB FID: 4526), family relationship satisfaction (UKB FID: 4559), friendships satisfaction (UKB FID: 4570), mental satisfaction (UKB FID: 4548), financial situation, and satisfaction (UKB FID: 4581). With the tolerance level of zero for all variables, except 1 for age and 0.5 for BMI, our case–control matching yielded a total number of 6,245 controls.

For other potential confounding reasons, we collected data on the prevalence of other common co-morbid conditions and reported the occurrence of each condition among both cases and controls (Table 1). These conditions included MS (ICD: G35), hydrocephalus (ICD: G91.0, G91.1, G91.3, G91.8, G91.9), epilepsy (ICD: G40-G41), sleep disorders (ICD: F51.0, F51.8, F51.9, G47.1, G47.8, G47.9, Z91.3), mental health disorders (ICD: F10-F99), dementia (ICD: F00-F02, G30-G32), brain tumors (ICD: C71), and cerebrovascular diseases (ICD: G46, I61, I63-I64, I67). However, for sample size reasons, and despite the distribution not being perfectly balanced between cases and controls, we did not exclude these conditions since exclusion would significantly reduce the number of participants.

**Table 1:** Demographic characteristics of the final sample populations of trigeminal neuralgia (TN) cases and controls

Variable	TN patients (n = 555)	Controls (n = 6245)
Age (mean ± SD)	59.1 ± 7.3	62.9 ± 4.7
Sex (female), n (%)	378 (68%)	4008 (64%)
Brain tumours (ICD: C71), n (%)	0 (0%)	8 (0.1%)
Cerebrovascular diseases (ICD: G46, I61, I63-I64, I67), n (%)	69 (12.4%)	304 (4.9%)
Dementia (ICD: F00-F02, G30-G32), n (%)	19 (3.4%)	110 (1.8%)
Epilepsy (ICD: G40-G41), n (%)	19 (3.4%)	88 (1.4%)
Hydrocephalus (ICD: G91.0, G91.1, G91.3, G91.8, G91.9), n (%)	6 (1.1%)	15 (0.2%)
Mental health disorders (ICD: F10-F99), n (%)	169 (30.5%)	884 (14.2%)
Multiple sclerosis (ICD: G35), n (%)	42 (7.6%)	23 (0.4%)
Sleep disorders (ICD: F51.0, F51.8, F51.9, G47.1, G47.8, G47.9, Z91.3), n (%)	0 (0%)	7 (0.1%)

### Statistical Analyses

Each SNP was coded as follows: (0) for homozygous for the major allele, (1) for heterozygous genotype, and (2) for homozygous for the minor allele. Using a case–control design, binary logistic regression models, adjusted for age, sex and first ten genetic principal components, were fit to compare the genotypes of TN cases with controls. Furthermore, sensitivity analyses were conducted for significant associations in the main analyses after excluding cases with TN as secondary diagnosis (n = 319). Thus, n = 236 cases were included in the sensitivity analyses. All analyses were conducted using Statistical Software for Social Sciences (SPSS) and R. To account for multiple comparisons, Bonferroni correction was applied and statistical significance level was set at P ≤ 0.00029 (0.05/175 based on the total number of SNPs included in the analysis).

### Genotype-Tissue Expression (GTEx) portal

To investigate the association between SNPs and gene expression, the public Expression Quantitative Trait Locus (eQTL) database, the Genotype-Tissue Expression (GTEx) portal, was searched. The GTEx Project was designed to enable studies of the relationships among genetic variation, gene expression, and other molecular phenotypes across multiple human tissues (GTex Consortium 2013). The database contains data on eQTLs derived from analyses performed in whole blood. Details on the eQTL analyses are explained both in other sources (GTex Consortium 2013) and on the GTEx website.

## Results

### Demographic Data

The demographic characteristics of the study sample population for both cases and controls are presented in Table 1. The study included 555 TN cases with a mean age of 59.1 years, comprising more females (n = 378, 68%) than males (n = 177, 32%). Controls (n = 6245) were slightly older (mean age: 62.9 years) and with similar distribution between females and males (4008 females vs. 2237 males, 64% vs. 36%) to cases. The occurrence of common co-morbid conditions was not perfectly balanced within cases and controls. For all conditions, the proportion was higher in cases compared to controls, except for sleep disorders and brain tumors, for which no cases were found among TN.

### Association Between SNPs and TN

We conducted binary logistic regression adjusted for age, sex and first ten genetic principal components, to investigate the difference in genotypes between TN patients and controls. Two SNPs, *C8B* rs706484 [odds ratio (OR) (95% confidence interval (CI)): 1.357 (1.158–1.590); p: 0.00016] and *MFG-E8* rs2015495 [OR (95% CI): 1.313 (1.134–1.521); p: 0.00028], were significantly associated with TN (Table 2). Additionally, 22 SNPs including *C8B* rs2281598, *C8B* rs72670362, *C8B* rs2269113, *C8B* rs582317, *C8B* rs614020, *C8B* rs2236217, *TRAIL/TNFSF10* rs231987, *FKBP5* rs9357201, *FKBP5* rs2766533, *FKBP5* rs9348981, *SFRP1* rs17574424, *APOA4* rs5104, *APOA4* rs5100, *APOA4* rs5092, *APOA1* rs7116797, *LGMN* rs4904981, *LGMN* rs1242108, *LGMN* rs1015033, *LGMN* rs68062833, *LGMN* rs1242102, *MFG-E8* rs10859, *MFG-E8* rs17202544, *MFG-E8* rs555006940, *MFG-E8* rs2271715, and *ANGPTL4* rs2278236 showed nominally significant association with TN (p for all < 0.05), and none of them remained significant after Bonferroni correction (as shown in Table S1).

**Table 2:** Significantly associated SNPs with TN

Chromosome	Gene	SNP	Reference allele	MAF	Odds ratio	Lower CI	Upper CI	P-value
Chr1	C8B	rs706484	T	0.398	1.357	1.158	1.59	0.00016
Chr15	MFG-E8	rs2015495	T	0.485	1.313	1.134	1.521	0.00028

This table shows the binary regression analysis of the association between SNPs and the TN phenotype. Analysis was adjusted for age, sex and first ten genetic principal components. P-values in bold indicate statistical significance (p-value < 0.05/175 = 0.00029). Abbreviations: CI = confidence interval, MAF = minor allele frequency, SNP = single nucleotide polymorphism, TN = trigeminal neuralgia

### Sensitivity Analysis

For further specificity, we excluded all the participants diagnosed with TN secondary to other health conditions. Thus, for the SNPs that showed significant associations, a sensitivity analysis was conducted by including cases with TN as the main diagnosis only (n_cases_ = 236) and adjusting the binary logistic regression models for age, sex, and the first ten genetic principal components. Only the two SNPs that were significantly associated with TN (*CB8* rs706484 and *MFG-E8* rs2015495) were tested. In the sensitivity analyses, *CB8* rs706484 was significantly associated with TN [OR (95% CI): 1.305 (1.035–1.646); p = 0.025], but *MFG-E8* rs2015495 was not [OR (95% CI): 1.166 (0.939–1.449); p = 0.165]. However, for both SNPs, the direction of effect was consistent with the main analyses (Table S2).

### Association Between Significant SNPs and Gene Expression

**Fig. 1:**
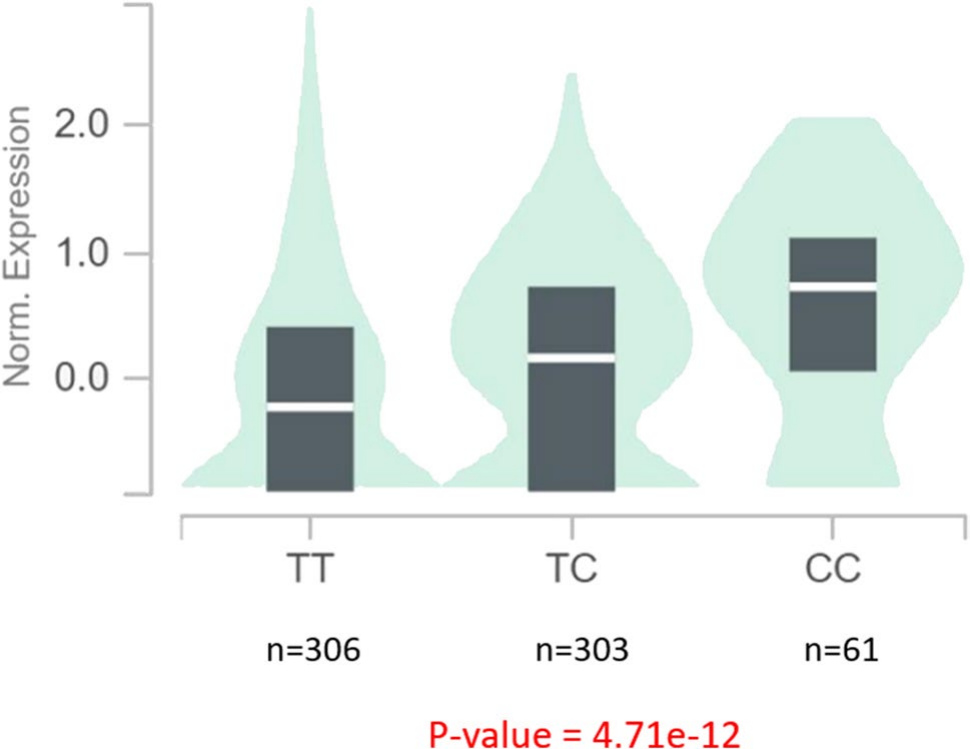
Violin plot of relative gene expression impacted by rs706484 eQTL of C8B gene (chromosome 1) in Whole Blood (GTEx portal). Each violin plot shows three expression distributions of the genotypes: homozygous reference (TT), heterozygous (TC), and homozygous alternative alleles (CC). T indicates the major/reference allele and C indicates the minor/risk allele with the number of subjects shown under each genotype. The plots indicate the density distribution of the samples in each genotype. The white line in the box plot (black) shows the median value of the gene expression at each genotype. The p-value is highlighted in red

**Fig. 2:**
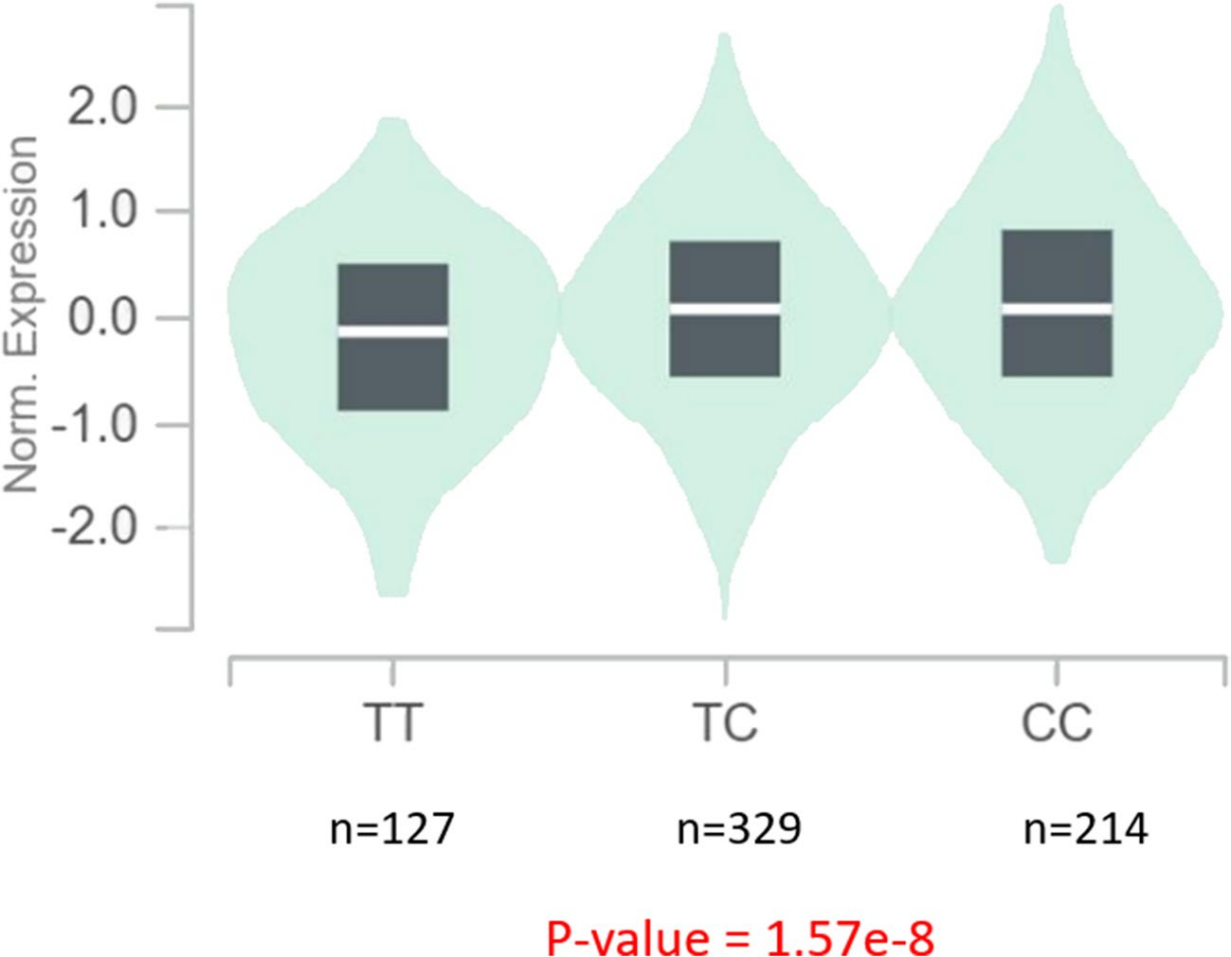
Violin plot of relative gene expression impacted by rs2015495 eQTL of MFG-E8 gene (chromosome 15) in Whole Blood (GTEx portal). Each violin plot shows three expression distributions of the genotypes: homozygous reference (TT), heterozygous (TC), and homozygous alternative alleles (CC). T indicates the major/reference allele and C indicates the minor/risk allele with the number of subjects shown under each genotype. The plots indicate the density distribution of the samples in each genotype. The white line in the box plot (black) shows the median value of the gene expression at each genotype. The p-value is highlighted in red

As SNPs have a wide distribution and can be found in any region of a gene, the SNPs that have functional implications on the levels of gene expression are called regulatory SNPs (rSNPs) or eQTLs, meaning they are associated with changes in the expression activity of their corresponding gene. The two uncovered significant SNPs were confirmed as true eQTLs based on data from whole blood in the public eQTL database GTEx portal (Fig. 1 and 2).

## Discussion

To our knowledge, this is the first study in the UKB focusing on TN and genetics. The frequency of TN observed in the UKB dataset aligns closely with the prevalence rates reported in prior research, which ranges between 0.1–0.7% (Mueller et al. 2011). In total, we identified 175 independent SNPs in the genes corresponding to the 17 proteins previously implicated in TN (Ericson et al. 2019, Abu Hamdeh et al. 2020, Svedung Wettervik et al. 2022, Lafta et al. 2023). This study suggests two novel SNPs, corresponding to the C8B and MFG-E8 genes, which demonstrate significant positive associations with TN, indicating a positive effect of the SNP alleles on gene expression and disease risk. This finding aligns with the expected biological effect, considering the involvement of these genes in the complement system and neuroinflammation, respectively, processes that are relevant to the pathophysiology of TN, as detailed below. The discovery of the two SNPs provides genetic evidence supporting previous proteomic findings for C8B (Abu Hamdeh et al. 2020) and MFG-E8 (Svedung Wettervik et al. 2022) and may help explain why some individuals develop TN while others do not, indicating a potential genetic predisposition to the condition.

The first candidate SNP, *C8B* rs706484, is located within the C8B gene, which is part of the eighth complement component (C8) subset, including alpha, beta, and gamma. The complement cascade, a crucial component of the complex innate immune surveillance system, plays a key role in the inflammatory response. Under normal conditions, complement components circulate as inactive precursors and can be cleaved into smaller active fragments through various proteolytic cascades. These fragments are essential for defending the body against pathogens by promoting phagocytosis or activating the adaptive immune system (Ricklin et al. 2016, Parker & Sodetz 2002). The complement system is part of the inflammatory response following nerve damage, with the complement component 8 gamma chain (C8G) specifically linked to neuroinflammation (Kim et al. 2021a, 2021b). A meta-analysis of 20 microarray studies on rodents with chronic pain models revealed that complement genes are among the most frequently and highly upregulated in both neuropathic and inflammatory pain conditions (LaCroix-Fralish et al. 2011). A rodent model with partial ligation of the sciatic nerve increased complement activity, contributing to inflammatory cell infiltration and neuropathic pain behaviors. Moreover, inhibiting complement activation by recombinant soluble human complement receptor type-1 reduced neuropathic pain development by suppressing T-cell and macrophage recruitment (Li et al. 2007). Although the characteristics of the pain model differ from TN, accumulating evidence suggests that complement components play a pivotal role in central nervous system pathology. This indicates a potential therapeutic strategy for neuroinflammatory diseases based on regulating their activation. However, successful clinical application requires a deeper and more extensive understanding of this cascade and the roles of complement components.

The other candidate SNP, *MFG-E8* rs2015495, corresponds to milk fat globule epidermal growth factor 8 (MFG-E8), a multifunctional glycoprotein initially discovered in the mammary epithelial cells of lactating mammary glands (Aoki et al. 1997). It is also found in various organs such as the heart, lungs, vessels, and the brain (Li et al. 2013). MFG-E8 has gained recognition for its diagnostic and prognostic implications in neurological degenerative disorders, including Alzheimer’s disease (Xu et al. 2018), Parkinson’s disease (Nakashima et al. 2018), and frontotemporal dementia (Campo et al. 2018). Produced by microglia and astrocytes, MFG-E8 plays a role in microglial phagocytosis of apoptotic cells by recognizing phosphatidylserine on these cells (Aziz et al. 2011). Additionally, MFG-E8 is involved in transforming macrophages into an anti-inflammatory phenotype, triggering the production of anti-inflammatory molecules such as tumor necrosis factor (TGF-β) and interleukin 10 (IL-10) (Shi et al. 2015). TNF-β, reported to be elevated in the CSF of TN patients (Ericson et al. 2019), contributes to the development of tertiary lymphoid organs, potentially reflecting the observed arachnoiditis associated with the trigeminal nerve in TN. Notably, tertiary lymphoid organs produce MFG-E8 (Krautler et al. 2012), and elevated MFG-E8 levels could potentially reflect such arachnoiditis. Previous studies also suggest that MFG-E8 mediates the phagocytic neuronal cell death observed during neuroinflammation (Fricker et al. 2012), suggesting that elevated MFG-E8 levels in TN patients may reflect trigeminal degeneration. Furthermore, MFG-E8 is implicated in vascular aging, promoting atherosclerosis development (Li et al. 2013). Classical TN, commonly occurring in older patients, may be linked to vascular aging and degeneration, as indicated by increased MFG-E8 levels.

Interestingly, both candidate SNPs were eQTLs, indicating their role in influencing gene expression variation, potentially influencing the biological pathways related to TN. The connection between genes and diseases is not always straightforward, and eQTLs can shed light on relationships between genetic variation and disease phenotypes. While GWAS play a crucial role, functional studies like the GTEx program (GTEx Consortium 2015) complement these efforts by identifying eQTLs that link SNP markers with mRNA expression (Westra & Franke 2014). It's essential to note that genetic variants may also impact protein levels through mechanisms not identifiable by eQTL analyses, involving post-transcriptional processes such as stability, translation, secretion, and/or detection of the gene product. Our discovery of SNPs functioning as eQTLs provides new insights bridging the gap between genomics and transcriptomics in the context of TN.

Some limitations of this study should be considered. The study is confined to the findings of four previous proteomic studies and includes a limited set of genes, 17 in total, and their associated SNPs. Consequently, there may be additional relevant genetic markers related to other genes that were not included. In addition, we focused on SNPs in the gene-coding regions only to make the analysis more targeted and less susceptible to the noise introduced by distant regulatory SNPs, which may have indirect or context-dependent effects on gene expression. However, this strategy limited the selection of variants and reduced the probability of detecting intergenic variants. Furthermore, binary regression typically analyzes one SNP at a time and may not effectively capture interactions between multiple genes or between genes and environmental factors. Although our sensitivity analysis with only primary diagnoses did not show a significant association for MFG-E8 rs2015495, the direction of effect was consistent with TN of both primary and secondary diagnoses. The non-significant result could be due to decreased statistical power owing to considerably small number of cases with primary TN diagnosis in the sensitivity sample compared to main study sample. To reduce confounding due to population substructure and ancestry heterogeneity, only data from European participants was included, which may limit the generalization of the findings to other populations and ancestries. For several of the covariates included in the matching process, they were self-reported, potentially reducing the reliability of the data. And lastly, the TN cases and controls were not perfectly balanced regarding various common co-morbid conditions, which for sample size reasons were not excluded, potentially confounding the results. However, despite these limitations, the study is the first one conducted on TN and genetics based on the UKB, and we identified interesting novel genetic biomarkers associated with gene expression that may be involved in the pathophysiology of TN.

## Conclusion

We have identified a novel association involving two independent genetic variants and TN, each corresponding to a different gene. The first SNP, *C8B* rs706484, is involved in the complement system, indicating its role in the neuroinflammatory process in TN. Additionally, we discovered rs2015495 in *MFG-E8*, a multifunctional glycoprotein known to potentially regulate the phagocytic death of neuronal cells during neuroinflammation. The identified genetic variations may help explain why some individuals develop TN while others do not, indicating a potential genetic predisposition to the condition. Furthermore, both of these genetic variants are expression eQTLs, implying that they could influence gene expression and thereby affect the biological pathways relevant to TN. In conclusion, genetic variations in genes that are part of the complement system and neuroinflammatory pathways may collectively contribute to an increased risk of developing TN.

## Supplementary Information

Below is the link to the electronic supplementary material.

Supplementary file1 (DOCX 51 KB)

## Data Availability

No datasets were generated or analysed during the current study.
