# Supplementary material for: Genomic Validation in the UK Biobank Cohort Suggests a Role of C8B and MFG-E8 in the Pathogenesis of Trigeminal Neuralgia
Source: J Mol Neurosci. 2024 Oct 3;74(4):91. doi: 10.1007/s12031-024-02263-x (PMC11449953; doi:10.1007/s12031-024-02263-x)
Supplement: Supplementary file 1 — Supplementary file1 (DOCX 51 KB) [file 12031_2024_2263_MOESM1_ESM.docx]

# Table S1: Binary regression analysis of the association between SNPs and trigeminal neuralgia (TN) phenotype.

| **Chromosome** | **Gene** | **SNP** | **Reference allele** | **MAF** | **Odds ratio** | **Lower CI** | **Upper CI** | **P-value** |
| --- | --- | --- | --- | --- | --- | --- | --- | --- |
| Chr1 | C8B | rs2281598 | C | 0.177 | 1.325 | 1.088 | 1.614 | **0.006** |
| Chr1 | C8B | rs72670362 | C | 0.17 | 0.781 | 0.653 | 0.935 | **0.008** |
| Chr1 | C8B | rs2269113 | C | 0.236 | 1.368 | 1.135 | 1.647 | **0.001** |
| Chr1 | C8B | rs582317 | G | 0.324 | 1.2 | 1.029 | 1.4 | **0.021** |
| Chr1 | C8B | rs614020 | G | 0.458 | 1.215 | 1.047 | 1.41 | **0.011** |
| Chr1 | C8B | rs2236217 | C | 0.23 | 1.411 | 1.138 | 1.748 | **0.002** |
| Chr1 | C8B | rs2795 | A | 0.368 | 0.967 | 0.825 | 1.133 | 0.676 |
| Chr1 | C8B | rs605648 | T | 0.12 | 0.868 | 0.695 | 1.084 | 0.213 |
| Chr1 | C8B | rs2025006 | C | 0.39 | 1.086 | 0.937 | 1.259 | 0.272 |
| Chr1 | C8B | rs597181 | G | 0.385 | 0.925 | 0.787 | 1.088 | 0.349 |
| Chr1 | C8B | rs72670363 | T | 0.155 | 0.847 | 0.71 | 1.011 | 0.067 |
| Chr1 | C8B | rs706482 | C | 0.246 | 0.856 | 0.726 | 1.01 | 0.066 |
| Chr1 | C8B | rs683916 | A | 0.352 | 0.97 | 0.828 | 1.136 | 0.704 |
| Chr1 | C8B | rs637253 | A | 0.352 | 0.86 | 0.729 | 1.014 | 0.073 |
| Chr1 | C8B | rs599857 | T | 0.335 | 0.904 | 0.761 | 1.074 | 0.253 |
| Chr1 | C8B | rs737097 | G | 0.165 | 0.894 | 0.743 | 1.074 | 0.231 |
| Chr1 | C8B | rs927213 | A | 0.129 | 0.838 | 0.697 | 1.008 | 0.061 |
| Chr1 | C8B | rs927211 | G | 0.344 | 0.925 | 0.783 | 1.091 | 0.354 |
| Chr1 | C8B | rs927210 | A | 0.489 | 1.127 | 0.972 | 1.306 | 0.115 |
| Chr1 | C8B | rs61767008 | A | 0.244 | 0.929 | 0.737 | 1.17 | 0.53 |
| Chr3 | TNFSF10 | rs231987 | G | 0.134 | 1.228 | 1.002 | 1.504 | **0.049** |
| Chr3 | TNFSF10 | rs11720451 | T | 0.324 | 1.089 | 0.934 | 1.269 | 0.275 |
| Chr3 | TNFSF10 | rs1131535 | C | 0.479 | 0.956 | 0.823 | 1.109 | 0.551 |
| Chr3 | TNFSF10 | rs3136605 | A | 0.329 | 0.926 | 0.773 | 1.11 | 0.408 |
| Chr3 | TNFSF10 | rs3136604 | G | 0.155 | 0.938 | 0.785 | 1.121 | 0.48 |
| Chr3 | TNFSF10 | rs3136602 | G | 0.2 | 1.007 | 0.841 | 1.206 | 0.94 |
| Chr3 | TNFSF10 | rs3821821 | C | 0.197 | 0.978 | 0.827 | 1.156 | 0.793 |
| Chr3 | TNFSF10 | rs7643953 | C | 0.21 | 1.04 | 0.879 | 1.23 | 0.648 |
| Chr3 | TNFSF10 | rs3136591 | G | 0.271 | 0.969 | 0.834 | 1.126 | 0.686 |
| Chr3 | TNFSF10 | rs231983 | T | 0.405 | 0.93 | 0.799 | 1.082 | 0.347 |
| Chr3 | TNFSF10 | rs3136589 | T | 0.255 | 0.933 | 0.763 | 1.142 | 0.503 |
| Chr3 | TNFSF10 | rs231980 | C | 0.325 | 0.964 | 0.81 | 1.147 | 0.679 |
| Chr3 | TNFSF10 | rs1823227 | T | 0.175 | 0.954 | 0.803 | 1.133 | 0.591 |
| Chr6 | LTA | rs1800683 | G | 0.39 | 1.031 | 0.887 | 1.198 | 0.691 |
| Chr6 | LTA | rs2239704 | A | 0.351 | 0.951 | 0.818 | 1.105 | 0.512 |
| Chr6 | LTA | rs2229094 | T | 0.275 | 1.044 | 0.885 | 1.232 | 0.611 |
| Chr6 | APOM | rs805297 | C | 0.249 | 0.989 | 0.838 | 1.166 | 0.892 |
| Chr6 | FKBP5 | rs9357201 | C | 0.364 | 1.172 | 1.006 | 1.365 | **0.042** |
| Chr6 | FKBP5 | rs2766533 | G | 0.367 | 1.181 | 1.018 | 1.37 | **0.029** |
| Chr6 | FKBP5 | rs9348981 | T | 0.26 | 0.818 | 0.691 | 0.968 | **0.02** |
| Chr6 | FKBP5 | rs1043805 | T | 0.197 | 1.103 | 0.907 | 1.342 | 0.327 |
| Chr6 | FKBP5 | rs3800373 | C | 0.326 | 1.12 | 0.955 | 1.313 | 0.165 |
| Chr6 | FKBP5 | rs7757037 | G | 0.473 | 0.931 | 0.803 | 1.08 | 0.348 |
| Chr6 | FKBP5 | rs11758051 | T | 0.405 | 0.891 | 0.746 | 1.064 | 0.202 |
| Chr6 | FKBP5 | rs3798346 | A | 0.121 | 1.036 | 0.872 | 1.231 | 0.687 |
| Chr6 | FKBP5 | rs737054 | G | 0.194 | 0.896 | 0.755 | 1.064 | 0.211 |
| Chr6 | FKBP5 | rs9470069 | G | 0.223 | 0.996 | 0.789 | 1.257 | 0.974 |
| Chr6 | FKBP5 | rs7760951 | T | 0.161 | 1.035 | 0.852 | 1.257 | 0.73 |
| Chr6 | FKBP5 | rs9394307 | A | 0.263 | 0.924 | 0.789 | 1.082 | 0.325 |
| Chr6 | FKBP5 | rs9368882 | A | 0.169 | 1.166 | 0.989 | 1.375 | 0.067 |
| Chr6 | FKBP5 | rs9470079 | G | 0.235 | 0.971 | 0.793 | 1.189 | 0.779 |
| Chr6 | FKBP5 | rs13215797 | C | 0.147 | 0.886 | 0.695 | 1.129 | 0.327 |
| Chr6 | FKBP5 | rs55987213 | C | 0.134 | 0.953 | 0.798 | 1.139 | 0.6 |
| Chr6 | FKBP5 | rs4711428 | C | 0.476 | 0.867 | 0.748 | 1.006 | 0.06 |
| Chr6 | FKBP5 | rs2766532 | A | 0.264 | 1.164 | 0.988 | 1.37 | 0.069 |
| Chr6 | FKBP5 | rs2766534 | G | 0.309 | 0.971 | 0.81 | 1.163 | 0.749 |
| Chr6 | FKBP5 | rs12200498 | G | 0.102 | 1.04 | 0.864 | 1.252 | 0.678 |
| Chr7 | PON1 | rs854550 | T | 0.154 | 0.987 | 0.819 | 1.19 | 0.895 |
| Chr7 | PON1 | rs854552 | C | 0.367 | 0.962 | 0.814 | 1.138 | 0.653 |
| Chr7 | PON1 | rs854555 | A | 0.456 | 0.953 | 0.815 | 1.113 | 0.542 |
| Chr7 | PON1 | rs2237582 | A | 0.45 | 0.93 | 0.788 | 1.097 | 0.389 |
| Chr7 | PON1 | rs3917550 | G | 0.116 | 0.911 | 0.722 | 1.148 | 0.428 |
| Chr7 | PON1 | rs3917542 | C | 0.349 | 0.928 | 0.772 | 1.116 | 0.43 |
| Chr7 | PON1 | rs576780277 | T | 0.129 | 1.131 | 0.861 | 1.486 | 0.378 |
| Chr7 | PON1 | rs2299257 | A | 0.462 | 0.947 | 0.812 | 1.104 | 0.486 |
| Chr7 | PON1 | rs143449312 | G | 0.448 | 1.075 | 0.918 | 1.259 | 0.37 |
| Chr7 | PON1 | rs2299259 | G | 0.302 | 0.934 | 0.787 | 1.108 | 0.432 |
| Chr7 | PON1 | rs854556 | C | 0.183 | 1.036 | 0.888 | 1.209 | 0.651 |
| Chr7 | PON1 | rs854558 | T | 0.32 | 1.058 | 0.901 | 1.243 | 0.492 |
| Chr7 | PON1 | rs854562 | C | 0.143 | 1.003 | 0.855 | 1.176 | 0.973 |
| Chr7 | PON1 | rs2272365 | A | 0.185 | 0.972 | 0.781 | 1.208 | 0.796 |
| Chr7 | PON1 | rs854566 | A | 0.191 | 0.97 | 0.798 | 1.178 | 0.757 |
| Chr7 | PON1 | rs3917490 | C | 0.348 | 0.992 | 0.854 | 1.151 | 0.914 |
| Chr7 | PON1 | rs2049649 | A | 0.479 | 1.073 | 0.914 | 1.261 | 0.388 |
| Chr7 | PON1 | rs2299261 | A | 0.291 | 1.06 | 0.898 | 1.252 | 0.49 |
| Chr7 | PON1 | rs854568 | G | 0.354 | 0.975 | 0.808 | 1.177 | 0.793 |
| Chr7 | PON1 | rs2299262 | C | 0.441 | 1.013 | 0.867 | 1.184 | 0.87 |
| Chr7 | PON1 | rs854569 | T | 0.43 | 0.972 | 0.811 | 1.166 | 0.763 |
| Chr7 | PON1 | rs2237583 | C | 0.302 | 1.043 | 0.885 | 1.23 | 0.612 |
| Chr7 | PON1 | rs854570 | C | 0.487 | 1.037 | 0.888 | 1.212 | 0.646 |
| Chr8 | SFRP1 | rs17574424 | C | 0.137 | 1.237 | 1.038 | 1.474 | **0.018** |
| Chr8 | SFRP1 | rs3242 | G | 0.205 | 0.971 | 0.828 | 1.138 | 0.717 |
| Chr8 | SFRP1 | rs1127379 | T | 0.488 | 1.019 | 0.878 | 1.183 | 0.807 |
| Chr8 | SFRP1 | rs11555201 | A | 0.197 | 0.966 | 0.827 | 1.128 | 0.66 |
| Chr8 | SFRP1 | rs7013565 | T | 0.423 | 0.883 | 0.733 | 1.063 | 0.188 |
| Chr8 | SFRP1 | rs4736960 | C | 0.455 | 0.999 | 0.857 | 1.165 | 0.995 |
| Chr8 | SFRP1 | rs7838918 | C | 0.465 | 1.043 | 0.895 | 1.216 | 0.587 |
| Chr8 | SFRP1 | rs6651363 | G | 0.216 | 0.994 | 0.853 | 1.158 | 0.935 |
| Chr8 | SFRP1 | rs10109536 | C | 0.396 | 1.004 | 0.864 | 1.167 | 0.955 |
| Chr8 | SFRP1 | rs17652488 | A | 0.293 | 1.083 | 0.93 | 1.261 | 0.306 |
| Chr8 | SFRP1 | rs10958671 | A | 0.216 | 0.829 | 0.672 | 1.023 | 0.081 |
| Chr8 | SFRP1 | rs10958672 | C | 0.348 | 1.132 | 0.976 | 1.312 | 0.101 |
| Chr8 | SFRP1 | rs34328499 | C | 0.192 | 0.984 | 0.833 | 1.164 | 0.854 |
| Chr8 | SFRP1 | rs9694405 | G | 0.446 | 1.131 | 0.949 | 1.348 | 0.168 |
| Chr8 | SFRP1 | rs7829419 | T | 0.323 | 1.058 | 0.905 | 1.237 | 0.478 |
| Chr8 | SFRP1 | rs7825952 | A | 0.41 | 0.994 | 0.831 | 1.188 | 0.946 |
| Chr8 | SFRP1 | rs13282702 | A | 0.467 | 0.984 | 0.85 | 1.138 | 0.825 |
| Chr9 | C5 | rs2075049 | C | 0.441 | 0.992 | 0.855 | 1.15 | 0.911 |
| Chr9 | C5 | rs4310279 | G | 0.227 | 0.982 | 0.832 | 1.159 | 0.831 |
| Chr9 | C5 | rs7026551 | A | 0.29 | 1.013 | 0.842 | 1.219 | 0.889 |
| Chr9 | C5 | rs2269066 | C | 0.18 | 0.947 | 0.743 | 1.208 | 0.661 |
| Chr9 | C5 | rs10760132 | C | 0.449 | 0.998 | 0.858 | 1.16 | 0.977 |
| Chr9 | C5 | rs1035029 | G | 0.313 | 0.991 | 0.85 | 1.156 | 0.91 |
| Chr9 | C5 | rs3764912 | A | 0.492 | 1.041 | 0.898 | 1.206 | 0.597 |
| Chr9 | C5 | rs2159776 | C | 0.462 | 0.968 | 0.836 | 1.122 | 0.668 |
| Chr6 | C5 | rs10985125 | G | 0.42 | 0.98 | 0.846 | 1.136 | 0.792 |
| Chr9 | C5 | rs10985126 | T | 0.242 | 1.048 | 0.869 | 1.264 | 0.624 |
| Chr9 | C5 | rs10818498 | A | 0.425 | 0.976 | 0.841 | 1.133 | 0.752 |
| Chr9 | C5 | rs7033790 | T | 0.181 | 0.929 | 0.777 | 1.11 | 0.419 |
| Chr9 | C5 | rs41308036 | G | 0.148 | 1.069 | 0.867 | 1.317 | 0.533 |
| Chr11 | APOA4 | rs5104 | C | 0.241 | 0.761 | 0.598 | 0.968 | **0.026** |
| Chr11 | APOA4 | rs5100 | G | 0.412 | 0.806 | 0.687 | 0.946 | **0.009** |
| Chr11 | APOA4 | rs5092 | C | 0.281 | 0.763 | 0.615 | 0.946 | **0.014** |
| Chr11 | APOA1 | rs7116797 | A | 0.359 | 0.736 | 0.571 | 0.949 | **0.018** |
| Chr11 | APOA1 | rs5070 | A | 0.447 | 0.976 | 0.83 | 1.148 | 0.769 |
| Chr11 | APOA1 | rs670 | C | 0.189 | 1.05 | 0.858 | 1.285 | 0.636 |
| Chr14 | LGMN | rs4904981 | G | 0.101 | 1.228 | 1.015 | 1.485 | **0.035** |
| Chr14 | LGMN | rs1242108 | C | 0.442 | 0.826 | 0.689 | 0.99 | **0.039** |
| Chr14 | LGMN | rs1015033 | C | 0.323 | 1.213 | 1.046 | 1.407 | **0.011** |
| Chr14 | LGMN | rs68062833 | G | 0.165 | 0.807 | 0.655 | 0.993 | **0.043** |
| Chr14 | LGMN | rs1242102 | C | 0.352 | 1.203 | 1.035 | 1.397 | **0.016** |
| Chr14 | LGMN | rs2250672 | T | 0.438 | 1.03 | 0.887 | 1.195 | 0.7 |
| Chr14 | LGMN | rs2402189 | A | 0.271 | 0.946 | 0.812 | 1.102 | 0.475 |
| Chr14 | LGMN | rs9791 | C | 0.282 | 0.956 | 0.822 | 1.11 | 0.553 |
| Chr14 | LGMN | rs4904977 | A | 0.497 | 1.028 | 0.886 | 1.192 | 0.715 |
| Chr14 | LGMN | rs6575282 | C | 0.314 | 0.925 | 0.792 | 1.082 | 0.33 |
| Chr14 | LGMN | rs12889434 | C | 0.321 | 0.957 | 0.822 | 1.115 | 0.574 |
| Chr14 | LGMN | rs3783933 | C | 0.342 | 1.074 | 0.9 | 1.282 | 0.429 |
| Chr14 | LGMN | rs1242118 | G | 0.225 | 0.994 | 0.851 | 1.161 | 0.944 |
| Chr14 | LGMN | rs1242116 | C | 0.405 | 0.967 | 0.83 | 1.126 | 0.664 |
| Chr14 | LGMN | rs28533172 | C | 0.401 | 0.913 | 0.782 | 1.066 | 0.249 |
| Chr14 | LGMN | rs4904980 | T | 0.337 | 0.942 | 0.78 | 1.137 | 0.535 |
| Chr14 | LGMN | rs1242112 | G | 0.113 | 0.983 | 0.842 | 1.149 | 0.834 |
| Chr14 | LGMN | rs1010651 | A | 0.277 | 0.875 | 0.75 | 1.022 | 0.092 |
| Chr14 | LGMN | rs3783930 | C | 0.195 | 0.979 | 0.796 | 1.204 | 0.84 |
| Chr14 | LGMN | rs1242105 | C | 0.176 | 1.07 | 0.909 | 1.259 | 0.417 |
| Chr14 | LGMN | rs17736427 | C | 0.364 | 0.873 | 0.744 | 1.025 | 0.099 |
| Chr14 | LGMN | rs2236264 | C | 0.234 | 0.872 | 0.715 | 1.064 | 0.177 |
| Chr14 | LGMN | rs1242101 | G | 0.199 | 1.06 | 0.907 | 1.238 | 0.465 |
| Chr14 | LGMN | rs7157038 | T | 0.393 | 0.885 | 0.755 | 1.039 | 0.136 |
| Chr14 | LGMN | rs79132724 | A | 0.245 | 0.908 | 0.761 | 1.084 | 0.285 |
| Chr14 | LGMN | rs1242100 | T | 0.278 | 0.977 | 0.785 | 1.214 | 0.832 |
| Chr14 | LGMN | rs1242095 | A | 0.44 | 0.912 | 0.772 | 1.078 | 0.281 |
| Chr15 | MFGE8 | rs10859 | G | 0.189 | 0.802 | 0.673 | 0.955 | **0.014** |
| Chr15 | MFGE8 | rs17202544 | C | 0.344 | 0.818 | 0.705 | 0.95 | **0.009** |
| Chr15 | MFGE8 | rs555006940 | A | 0.261 | 0.754 | 0.626 | 0.91 | **0.004** |
| Chr15 | MFGE8 | rs2271715 | G | 0.365 | 1.251 | 1.075 | 1.455 | **0.004** |
| Chr15 | MFGE8 | rs8530 | C | 0.117 | 0.845 | 0.66 | 1.081 | 0.18 |
| Chr15 | MFGE8 | rs2280215 | C | 0.344 | 0.887 | 0.755 | 1.043 | 0.148 |
| Chr15 | MFGE8 | rs4932449 | G | 0.375 | 0.921 | 0.782 | 1.086 | 0.33 |
| Chr15 | MFGE8 | rs4932450 | G | 0.244 | 0.936 | 0.78 | 1.125 | 0.482 |
| Chr15 | MFGE8 | rs28384224 | T | 0.417 | 1.051 | 0.896 | 1.234 | 0.54 |
| Chr15 | MFGE8 | rs2280213 | T | 0.205 | 0.976 | 0.822 | 1.16 | 0.784 |
| Chr15 | MFGE8 | rs3784751 | A | 0.424 | 0.91 | 0.776 | 1.067 | 0.247 |
| Chr15 | MFGE8 | rs34239095 | C | 0.2 | 0.959 | 0.813 | 1.131 | 0.621 |
| Chr15 | MFGE8 | rs35936125 | C | 0.262 | 0.943 | 0.805 | 1.105 | 0.467 |
| Chr19 | ANGPTL4 | rs2278236 | G | 0.421 | 1.192 | 1.03 | 1.38 | **0.019** |
| Chr19 | ANGPTL4 | rs12609364 | G | 0.205 | 1.064 | 0.902 | 1.256 | 0.463 |
| Chr19 | ANGPTL4 | rs112711963 | T | 0.102 | 1.046 | 0.863 | 1.267 | 0.649 |
| Chr19 | ANGPTL4 | rs1044250 | C | 0.24 | 0.92 | 0.783 | 1.08 | 0.307 |
| Chr19 | TBCB | rs10425700 | G | 0.216 | 1.059 | 0.898 | 1.249 | 0.497 |
| Chr19 | TBCB | rs10407011 | G | 0.259 | 1.052 | 0.897 | 1.233 | 0.536 |
| Chr19 | TBCB | rs2072606 | C | 0.364 | 1.067 | 0.916 | 1.241 | 0.406 |
| Chr19 | TBCB | rs8104047 | G | 0.408 | 1.015 | 0.869 | 1.184 | 0.854 |
| Chr19 | TBCB | rs10410926 | G | 0.169 | 0.965 | 0.782 | 1.191 | 0.74 |
| Chr19 | TBCB | rs6510521 | G | 0.468 | 0.993 | 0.854 | 1.154 | 0.923 |
| Chr19 | APOC2 | rs9304644 | C | 0.443 | 0.989 | 0.852 | 1.148 | 0.884 |
| Chr19 | APOC2 | rs12721076 | T | 0.117 | 1.079 | 0.876 | 1.328 | 0.476 |
| Chr19 | APOC2 | rs9304645 | G | 0.224 | 1.116 | 0.927 | 1.344 | 0.245 |
| Chr19 | APOC2 | rs1130742 | C | 0.329 | 0.915 | 0.774 | 1.082 | 0.302 |
| Chr19 | CLEC11A | rs11084024 | G | 0.152 | 1.035 | 0.872 | 1.229 | 0.695 |
| Chr19 | CLEC11A | rs13866 | C | 0.276 | 0.946 | 0.797 | 1.123 | 0.527 |
| Chr19 | CLEC11A | rs1053020 | T | 0.326 | 1.053 | 0.891 | 1.245 | 0.547 |

This table shows the binary regression analysis of the association between SNPs and the TN phenotype. The analysis was adjusted for age, sex, and the first ten genetic principal components. All associations were non-significant after correction for multiple comparisons, although some were nominally significant (p-value < 0.05) with p-values in bold. Abbreviations: TN = trigeminal neuralgia, SNP = single nucleotide polymorphism, CI = confidence interval, MAF = minor allele frequency.

# Table S2: Sensitivity analysis on the association between primary TN and significant SNPs.

| **Chromosome** | **Gene** | **SNP** | **Reference allele** | **MAF** | **Odds ratio** | **Lower CI** | **Upper CI** | **P-value** |
| --- | --- | --- | --- | --- | --- | --- | --- | --- |
| Chr1 | C8B | rs706484 | T | 0.398 | 1.305 | 1.035 | 1.646 | **0.025** |
| Chr15 | MFGE8 | rs2015495 | T | 0.485 | 1.166 | 0.939 | 1.449 | 0.165 |

This table shows sensitivity analysis using binary regression models on the association between significant SNPs and the TN phenotype. Analysis was adjusted for age, sex and first ten genetic principal components. Only the two SNPs that were significantly associated with TN (both primary and secondary diagnoses) were tested. P-values in bold indicate statistical significance (p-value<0.05/2=0.025). Abbreviations: TN = trigeminal neuralgia, SNP = single nucleotide polymorphism, CI = confidence interval, MAF = minor allele frequency.
